# Antibiofilm Activity of Curcumin and Piperine and Their Synergistic Effects with Antifungals against *Candida albicans* Clinical Isolates

**DOI:** 10.1155/2024/2025557

**Published:** 2024-02-28

**Authors:** Ulrich Joël Tsopmene, Christian Ramsès Tokam Kuaté, Prudence Ngalula Kayoka-Kabongo, Borel Ndezo Bisso, Anisel Metopa, Clautilde Teugwa Mofor, Jean Paul Dzoyem

**Affiliations:** ^1^Department of Biochemistry, Faculty of Science, University of Dschang, Dschang, Cameroon; ^2^Department of Agriculture and Animal Health, College of Agriculture and Environmental Sciences, University of South Africa, Florida, South Africa; ^3^Department of Biochemistry, Faculty of Science, University of Yaoundé I, Yaoundé, Cameroon

## Abstract

**Background:**

Candidiasis is the common name for diseases caused by yeast of the genus *Candida*. *Candida albicans* is one of the most implicated species in superficial and invasive candidiasis. Antifungals, polyenes, and azoles have been used to treat candidiasis. However, due to the development of antifungal resistance, research of natural substances with potential antifungal effects at low concentrations or combined is also a possibility.

**Methods:**

The broth microdilution method was used to evaluate the antifungal activity. The biofilm formation was assessed using the microtiter plate method. The antibiofilm activities were assessed using micro plaque tetrazolium salt assay (MTT). The combination effect of antifungal with natural substances was made using the checkerboard method.

**Results:**

Among our isolates, clotrimazole was the most resistant, but amphotericin B was the most effective antifungal. The biofilm was formed by all isolates of *C. albicans*. Curcumin and piperine displayed antibiofilm activity with minimum biofilm inhibitory concentration (MBIC) and minimum eradicating concentration (MBEC) ranging from 64 to 1024 *μ*g/mL and 256 to 2048 *μ*g/mL. In combination, piperine presented double synergistic effects compared to curcumin with all antifungals tested. Curcumin shows more synergistic effect when combined with polyenes than with azoles. However, piperine shows a more synergistic effect when combined with azoles compared to polyenes.

**Conclusion:**

*C. albicans* was susceptible to curcumin and piperine both on planktonic cells and biofilm. The combination of curcumin and piperine with antifungals has shown synergistic effects against multiresistant clinical isolates of *Candida albicans* representing an alternative drug research for the treatment of clinical candidiasis.

## 1. Introduction

Fungal diseases have emerged and have been increasingly recognized as important public health problems owing to an ever-expanding population of immune-compromised patients. They are usually mostly caused by *Candida* species as *C. albicans* has been reported as the most prevalent pathogen in systemic fungal infections during the last three decades [1]. Antifungals are currently used in the treatment of yeast infections. Different antifungals are commonly used in therapy and target fungi: chitin synthesis, ergosterol synthesis, glucan synthesis, squalene epoxidase, nucleic acid synthesis, protein synthesis, and microtubule synthesis. Azoles (fluconazole and clotrimazole) are used to treat fungal infection and have fungistatic effects on *C. albicans* because they inhibit cytochrome P450 14*α*-lanosterol demethylase and, then, block the synthesis of ergosterol in the cytoplasm. Azoles reduce the amount of ergosterol in the membrane by inhibiting its synthesis in the cytosol. Antifungal polyenes such as amphotericin B and nystatin bind to ergosterol (the major sterol of the fungal membrane) and have fungicidal activities.

So, commercial antifungal agents, including fluconazole and amphotericin are widely prescribed, but they are not very effective in clinical situations [2]. Due to the toxicity of commercial antifungals and the multiresistance of *C. albicans* to antifungals, antifungal therapy to combat candidiasis is still ineffective [3]. The pathogenicity of *C. albicans* increases because of the resistance activity of virulence factors like biofilm formation and yeast-to-hyphae transition [2]. Biofilm is defined as a microbial community containing a dense network of yeast and filaments embedded inside an exopolymeric matrix that hinders the action of antimicrobials. It acts as a diffusion barrier against antifungals and holds immune factors in comparison to planktonic cells [4]. *C. albicans* biofilm shows increased resistance against most antifungal agents and is difficult to eradicate [2]. The increased cost and drug resistance have put limitations on the use of antifungal drugs, so there is a need to find better drug agents to cure life-threatening infections associated with the biofilm of *C. albicans* [2].

Among the potential sources of new agents, a new strategy consisting of the use of natural products to promote health is as old as human civilization. Recently, it was reported that natural products derived from plants as abundant sources of biologically active compounds have driven their exploitation toward the search for new chemical products that can lead to further pharmaceutical formulations [4]. Many studies have reported the in vitro activities of various yeast species. Curcumin, found in *Curcuma longa*, is an important Asian spice used in many food preparations. Previous studies report that curcumin is a promising anticandida compound of clinical interest [5]. Piperine is a naturally occurring alkaloid found in consumed species of black pepper (*Piper nigrum*) and long pepper (*Piper longum*) and has antimicrobial and antibiofilm activities against bacteria strains [6, 7].

Another approach to overcome microbial infections associated with biofilm formation is to use a combination therapy of natural substances with commercial antimicrobial drugs to enhance treatment [7]. Combination therapy is considered an effective approach to improving the efficacy of therapy in the treatment of invasive infections. Additionally, combination therapy is very useful and effective since it may increase both the rate and degree of microbial killing because each drug has a different mechanism of action [2]. Due to different targeting approaches, the development of drug resistance can be slowed down, and the liver toxicity of antifungals like fluconazole should be avoided with the help of two or more combined drugs [2]. This study aimed to evaluate the in vitro antifungal and antibiofilm activity of two natural substances, piperine (alkaloid) and curcumin (a polyphenol), and their combination with current antifungals, revealing species with inhibition/reduction effects on the biofilm formation in *Candida albicans* isolates.

## 2. Materials and Methods

### 2.1. Microorganisms and Cultures

The twenty clinical isolates of *C. albicans* used in this study were named: *Ca01*, *Ca02*, *Ca03*, *Ca04*, *Ca05*, *Ca06*, *Ca07*, *Ca08*, *Ca09*, *Ca10*, *Ca11*, *Ca12*, *Ca13*, *Ca14*, *Ca15*, *Ca16*, *Ca17*, *Ca18*, *Ca19*, and *Ca20*, and one reference strain ATCC 9002. These isolates were obtained from the Research Unit of Microbiology and Antimicrobial Substances (RUMAS) in the Faculty of Science of the University of Dschang, Cameroon. Sabouraud dextrose agar (SDA) (Liofilchem Laboratories) was used for the maintenance and culture of fungal strains, Sabouraud dextrose broth (SDB) (Liofilchem Laboratories) was used for the determination of the minimum inhibitory concentrations (MICs).

### 2.2. Chemicals

Antifungals: polyenes (amphotericin B and nystatin) and azoles (fluconazole and clotrimazole) were used. Natural compounds such as piperine (purity 97%) and curcumin (purity 65%) were also used. Tetrazolium salt assay (MTT) and dimethyl sulfoxide (DMSO, p-iodonitrotetrazolium chloride (INT) and Roswell Park Memorial Institute (RPMI-1640) medium, were used. All those chemicals were purchased from Sigma-Aldrich.

### 2.3. Antifungal Susceptibility

The minimum inhibitory concentrations (MICs) of the antifungals and natural products were determined by the method previously described [8]. The natural substances and antifungals were prepared at 4096 *μ*g/mL and 512 *μ*g/mL, respectively, and serially diluted twice with SDB in a 96-well microplate to obtain a final volume of 100 *μ*L. The concentrations of natural substances and antifungals ranged, respectively, from 2048 to 1 *μ*g/mL and 256 to 0.125 *μ*g/mL. Subsequently, 100 *μ*L of fungal inoculum at a concentration of 1.5 × 10^4^ CFU/mL was added to the microplate wells and incubated at 37°C for 48 hours. Wells containing only fungal inoculum represented the negative control; however, wells containing microorganisms and standard drugs were considered the positive control.

After incubation, the MIC endpoint was considered the lowest concentration of natural substances or antifungals where no growth was observed in the microplate. The use of vital dyes in assessing the antifungal activity of natural substances may compromise the comparability of the data.

The antifungal activity of natural products was considered as follows: most active (MIC value ≤1 *μ*g/mL), significant activity (1 ≤ MIC value ≤10 *μ*g/mL), moderate (10 ≤ MIC value ≤100 *μ*g/mL), and inactive (100 < MIC value ≤1000 *μ*g/mL) [9]. The cut-off values of antifungals previously described were used for *Candida albicans* [8]. For fluconazole, yeast with a MIC value ≤8 *μ*g/mL was considered susceptible, while yeast with 32 ≥ MIC value ≥32 *μ*g/mL was considered as intermediate, and yeast with a MIC value ≥64 *μ*g/mL was considered as resistant. For amphotericin B and nystatin, the MIC value ≤ 1 *μ*g/mL indicated that the yeast was susceptible, while yeast with 2 ≥ MIC value ≥4 *μ*g/mL was considered intermediate, and then, MIC value >4 *μ*g/mL indicated resistance. For clotrimazole, the MIC value ≤0.5 *μ*g/mL indicated that the yeast was susceptible, while yeast with 1 ≥ MIC value ≥2 *μ*g/mL was considered as intermediate, and a MIC value ≥4 *μ*g/mL means that the yeast was resistant.

### 2.4. Biofilm Formation Assay

The biofilm ability of *C. albicans* was determined by the microtiter plate assay method as previously described [10] with some modifications. In brief, 150 *μ*L of RPMI-1640 and 50 *μ*L of inoculum (1.5 ×  10^4^ CFU/mL) were introduced into a 96-well flat-bottomed sterile polystyrene microplate and incubated at 37°C for 48 hours. After incubation, planktonic cells in the well of the microplate were discharged by washing twice with 200 *μ*L of phosphate-buffered saline (PBS) at 7.2 pH. To perform biofilm formation, the MTT (tetrazolium salt 3-[4,5-dimethylthiazol-2-yl]-2,5-diphenyltetrazolium bromide, Sigma-Aldrich, USA) reduction assay was used. Briefly, 200 *μ*L of 0.5 mg/mL of MTT reagent prepared in PBS was introduced into each well of microplates and incubated at 37°C for 4 hours. Unincubated, well-stained, sterile RPMI-1640 was considered the negative control and was used as a blank. After incubation, the MTT solution was aspired, and 150 *μ*L of DMSO was introduced. The optical density (OD) of each well of the microplate was measured spectrophotometrically at 570 nm by using a microplate reader (VERSA-max). The ATCC 9002 stain was considered a positive control, while those containing only DMSO were considered blank. The percentage of biofilm formation was calculated using the formula described:

(1) $$\begin{array}{c} \%\text{ Biofilm formation}=\left[\frac{\left({\text{OD}}_{\text{test}}-{\text{ OD}}_{\text{blank}}\right)}{\left({\text{OD}}_{\text{control}}-{\text{ OD}}_{\text{blank}}\right)}\right]\times 100. \end{array}$$

### 2.5. Biofilm Inhibition Assay

The biofilm inhibition activity of curcumin, piperine, and antifungals was carried out according to the method previously described [11]. Briefly, 20 *μ*L of fungal inoculum (1.5 × 10^4^ CFU/mL) and 180 *μ*L of concentrations of antifungals or natural substances were introduced into the microplate. Final concentrations of antifungals and natural products, respectively, range from 8 to 1024 *μ*g/mL and 16 to 2048 *μ*g/mL, and the microplate was incubated at 37°C for 48 h. Then, the microplates then carefully cleared of their contents and washed three times with phosphate buffer (PBS), pH 7.2. A volume of 150 *μ*L of methanol was added to the well for biofilm fixation and removed after 15 min, and then 150 *μ*L of crystal violet (1%) was added for staining. Then, microplates were washed twice with PBS to discharge the stain. After the air-dying process, the dye of biofilms that lined the walls of the microplate was solubilized with 150 *μ*L of 98% ethanol. Then, the optical density (OD) of the microplate was measured spectrophotometrically at 570 nm by using a microplate reader. The study was performed three times. Uninoculated well containing sterile RPMI-1640 was used. The percentage of biofilm was calculated using the formula below, and the minimal biofilm inhibitory concentration (MBIC) was recorded as the lowest concentration of antifungals or natural substances that inhibit 100% of biofilm.

(2) $$\begin{array}{c} \%\text{ Biofilm inhibition activity}=\frac{\left({\text{OD}}_{\text{control}}-{\text{OD}}_{\text{blank}}\right)-\left({\text{OD}}_{\text{test}}-{\text{OD}}_{\text{blank}}\right)}{{\text{OD}}_{\text{control}-{\text{OD}}_{\text{blank}}}}\times 100. \end{array}$$

### 2.6. Biofilm Eradication Assay

The determination of the biofilm eradication potential of curcumin, piperine, and antifungals was performed as previously described [11]. Briefly, 200 *μ*L of fungal inoculum (1.5 × 10^4^ CFU/mL) and 180 *μ*L of RPMI-1640 were introduced into the microplate and incubated at 37°C for 48 h. Once the biofilm had formed, the microplate well was gently cleared of its contents and washed three times with PBS buffer. Then, 200 *μ*L of antifungals and natural substances at concentrations ranging from 8 to 1024 *μ*g/mL and 16 to 2048 *μ*g/mL and incubated at 37°C for 48 hours. After incubation, the microplate was treated as described previously for the biofilm inhibition assay. The test was repeated three times, and the percentage of biofilm eradication was calculated using the formula below. The minimal biofilm eradicating concentration (MBEC) was recorded as the lowest concentration of antifungals or natural substances that reduce 100% of biofilm.

(3) $$\begin{array}{c} \%\text{ Biofilm eradicating activity}=\frac{\left({\text{OD}}_{\text{control}}-{\text{OD}}_{\text{blank}}\right)-\left({\text{OD}}_{\text{test}}-{\text{OD}}_{\text{blank}}\right)}{{\text{OD}}_{\text{control}-{\text{OD}}_{\text{blank}}}}\times 100. \end{array}$$

### 2.7. Combination of Antifungals with Curcumin and Piperine against Planktonic *C. albicans* Isolates

The checkerboard assay as previously described [12] was used for the determination of the combined effects of antifungals with curcumin and piperine against *Candida albicans*. Briefly, 50 *μ*L of Sabouraud dextrose broth (SDB) was distributed into each well of the microdilution plates. Antifungals were serially diluted along the abscissa, and natural substances were serially diluted along the ordinate. Then, 100 *μ*L of fungal inoculum (1.5 × 10^4^ CFU/mL) was added to each well, and the well was incubated at 37°C for 48 h. The final concentration ranges from 0.25 to 256 *μ*g/mL for antifungals, 4–512 *μ*g/mL for curcumin, and 8–512 *μ*g/mL for piperine. After incubation, a volume of 40 *μ*L of INT (iodonitrotetrazolium chloride) was added to microplate wells and incubated at 37°C for 30 minutes. Viable fungal cells change the yellow dye of INT to a pink color. The minimum inhibitory concentrations (MICs) were considered the lowest natural product concentration that prevented the color change medium. The fractional inhibitory concentration index (ICIF) was calculated as follows: ICIF = (MIC of antifungal in combination/MIC of antifungal alone) + (MIC of a natural substance in combination/MIC of antifungal alone). FICI was interpreted as previously described [12]: synergy when FICI ≤ 0.5; additivity when 0.5 < FICI ≤ 1; indifference when 1 < FICI ≤ 4; and antagonism when FICI > 4.

### 2.8. Statistical Analysis

Statistical analysis was performed using GraphPad Prism version 8.0 for biofilm formation. The synergistic combinations of natural substances and antifungals were analyzed by using Microsoft Excel 2016.

## 3. Results

### 3.1. Antifungal Activities of Natural Substances and Antifungals

The susceptibility profile of *C. albicans* planktonic cells to antifungals (amphotericin B, nystatin, clotrimazole, and fluconazole) and natural substances is shown in Table 1. MIC values range from 0.125 to 256 *μ*g/mL and from 32 to 1024 *μ*g/mL for curcumin and piperine, respectively. The minimum inhibitory concentration values of antifungals ranged from 0.125 to 64; 0.25 to 128; 0.125 to 64; and 0.5 to 128 *μ*g/mL, respectively, for antifungals: amphotericin B, nystatin, clotrimazole, and fluconazole. According to the epidemiological cut-off values of antifungals, azoles (clotrimazole and fluconazole) were more resistant than polyenes (nystatin and amphotericin B). Clotrimazole was the antifungal agent with the highest frequency of resistance compared with the others. About natural substances, curcumin presented a significant activity with MICs of 0.125 and 8 *μ*g/mL, respectively, against *Ca07* and *Ca20*. Additionally, curcumin presented moderate activity, ranging from 16 to 64 *μ*g/mL. Moreover, piperine showed moderate activity with MICs ranging from 32 to 64 *μ*g/mL.

### 3.2. Biofilm Formation

**Figure 1:**
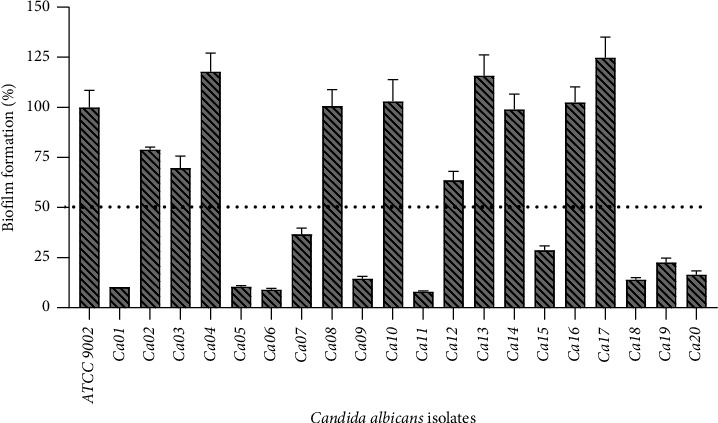
Percentage of biofilm formation ability by clinical isolates of *C. albicans* compared to reference strain ATCC 9002.

The biofilm formation kinetics was performed at 48 hours, and the mean optical density values were read at 570 nm. The percentage of biofilm formation was calculated compared to the biofilm formation of the reference strain ATCC 9002 and presented in Figure 1. The results showed that all our isolates formed biofilm at 48 hours with different percentages. The percentages of biofilm in the isolates (*Ca04*, *Ca08*, *Ca10*, *Ca13*, *Ca14*, *Ca16*, and *Ca*17) were more than for the reference strain ATCC 9002. The isolates *Ca02*, *Ca03*, *Ca10*, *Ca13*, *Ca14*, and *Ca16* presented a percentage of biofilm of more than 50%, and those who were resistant to more than one antifungal of the classes (polyenes and azoles) tested in this study were selected for the antibiofilm and combinations assay.

### 3.3. Antibiofilm Activities of Antifungals and Natural Substances

The antibiofilm activity, minimum biofilm inhibition concentration (MBIC), and minimum biofilm eradication concentration (MBEC) of the natural substances and antifungals, as well as the MBIC/MBIC ratio, are determined and presented in Table 2. The MBEC/MBIC ratio demonstrates the increased resistance in preformed biofilm compared to inhibitory biofilm formation. Curcumin showed better activity against *C. albicans* biofilm than piperine, with MBIC and MBEC values ranging from 64 to 1024 *μ*g/ml and 256 to 2048 *μ*g/ml, respectively. Antifungals showed MBIC values ranging from 16 to 1024 *μ*g/mL, 16–512 *μ*g/mL, 18–128 *μ*g/mL, and 16–512 *μ*g/mL for amphotericin B, nystatin, clotrimazole, and fluconazole, respectively. However, their MBEC values, respectively, ranged from 128 to 256 *μ*g/mL, 64–256 *μ*g/mL, 256–512 *μ*g/mL, and 256–1024 *μ*g/mL for amphotericin B, nystatin, clotrimazole, and fluconazole. According to the R (MBEC/MBIC) ratio, the concentration of antifungal or natural substances for inhibition was lower than that of the eradicated biofilm of *Candida albicans*.

### 3.4. A Combination of Effects of Curcumin and Piperine with Azoles and Polyenes against Clinical Isolates of *Candida albicans*

The effect of a combination of antifungals and natural substances was evaluated, and the results are presented in Table 3. In a combination study, the fractional inhibitory concentration index (FICI) was used to appreciate the interaction between natural products and antifungals. Curcumin combined with nystatin showed three synergistic effects against isolates and strains: ATCC 9002, *Ca13*, and *Ca14*, with respective FIC values of 0.5, 0.26, and 0.5. Curcumin reduced 4-fold, 64-fold, and 4-fold, respectively, the MIC of nystatin. Curcumin combined with amphotericin B showed three synergistic effects against isolate *Ca14* with respective FICI values of 0.5 and reduced 4-fold the MIC of amphotericin B. Two synergistic effects were also obtained with a combination of curcumin and fluconazole against isolates *Ca02* and *Ca03* with respective FICI values of 0.31 and 0.28, reducing 8-fold and 32-fold the MIC of fluconazole, respectively. Curcumin combined with amphotericin B showed one synergistic effect against *Ca14* with a FICI value of 0.5 and reduced 4-fold the MIC value of amphotericin B. No synergistic effects were obtained with a combination of curcumin and clotrimazole.

Piperine in combination with fluconazole showed five synergistic effects (FIC = 0.15 to 0.5) against *C. albicans* isolates *Ca10*, *Ca02*, *Ca14*, *Ca16*, and ATCC 9002 with a reduction of the MIC value of fluconazole (2048-, 256-, 2048-, and 512, respectively). Four synergistic effects were obtained with a combination of piperine and clotrimazole against isolates *Ca03*, *Ca02*, *Ca14*, and ATCC 9002 with FICI values (0.28, 0.37, 0.37, and 0.31, respectively) and reducing MIC values of fluconazole 32-fold, 4-fold, 8-fold, and 16-fold. Three synergistic effects were also reported with piperine and nystatin against isolates *Ca16*, *Ca14*, and ATCC 9002, with FIC values ranging from 0.15 to 0.37 and reducing MIC values of nystatin from 8 to 64-fold. Two synergistic effects were shown with piperine and amphotericin B against isolates *Ca02* and *Ca14* with FICI values 0.25 and 0.5, respectively, and reducing 8-fold and 4-fold the MIC value of amphotericin B.

Generally, in all our isolates and strains, piperine presented a double number (thirteen) of synergistic effects compared to curcumin (six) in combination with all antifungals. Curcumin presented more synergistic effects (four) combined with polyenes (amphotericin B and nystatin) compared to azoles (two) (clotrimazole and fluconazole). However, piperine presented more synergistic effects combined with azoles (nine) compared to polyenes (five).

## 4. Discussion

*C. albicans* is one of the most common pathogenic fungi in humans, causing superficial and systemic infections. The ability of *C. albicans* to form biofilms makes them resistant and more tolerant to antimicrobial therapy. Given the resistance of *C. albicans* to antifungal agents as a result of biofilm formation, it is becoming difficult to predict which molecules will emerge as new clinical antifungal agents. Biofilm formation makes treatment difficult and contributes to high rates of morbidity and mortality. Current antifungals are extremely limited, and six classes of antifungal drugs are used to treat fungal infections, namely, azole derivatives, polyenes, echinocandins, 5-fluorocytosine, allylamines, and morpholines [13].

The antifungal susceptibility of *C. albicans* against antifungals and natural substances is presented in Table 1. Clotrimazole was the antifungal agent with the highest incidence of resistance. However, amphotericin B was the most effective against *C. albicans*. Comparatively, previous studies show the MIC values range from 1 to 16 *μ*g/mL for nystatin and a MIC value of 0.5 *μ*g/mL for amphotericin B [14]. Moreover, it was reported that MIC values for clotrimazole ranged from 8 to 16 *μ*g/mL and from 32 to 64 *μ*g/mL. Clotrimazole was reported as the most effective anticandida drug compared to fluconazole and nystatin [15]. Our results corroborated those obtained previously. According to the epidemiological cut-off values of antifungals, azoles were more resistant than polyenes. Compounds that act by lysing the membrane have lower resistance rates. This is because the modifications to the plasma membrane induced by the pathogen to become resistant to these compounds normally have a major impact on its viability. For their mechanism of action, azoles (fluconazole and clotrimazole) act on the inhibition of lanosterol 14 *α*-demethylase (ERG11; ergosterol biosynthesis), and polyenes (nystatin and amphotericin B) bind to ergosterol in the fungal cell membranes; formation of transmembrane pores, resulting in loss of membrane integrity, and interruption of the ion gradient, and disturbing normal membrane function. This high resistance of *Candida* strains to azoles may be caused by drug efflux due to a reduction in the affinity of the Erg11 protein through mutations. Mutations in the Erg11 protein also upregulate multiple drug transporter genes. Changes in specific stages of the ergosterol biosynthesis pathway were seen [13].

Due to the development of the resistant form of *Candida albicans*, conventional drugs can be sometimes ineffective. Herbs and naturally imitative bioactive compounds could be a new source of antimycotic therapy. Several review studies suggest that herbal medicines and natural bioactive compounds have antifungal effects [16]. Nutraceuticals such as curcumin (*Curcuma longa*, polyphenol) and piperine (*Piper nigrum* and *Piper longum* an alkaloid) are useful in the treatment of *C. albicans* in candidiasis and could be a safe, accessible, and inexpensive management option to prevent and treat disease [16, 17].

The anti-*C. albicans* susceptibility to curcumin and piperine was evaluated and presented in Table 1. Curcumin presented significant and moderate activities. Moreover, piperine shows moderate activity on *C. albicans* isolates. Our results corroborate the previous studies reporting that curcumin and piperine were inactive against the majority of *C. neoformans* fungus isolates with MIC values of more than 100 *μ*g/mL. Compounds with a lytic action on the membrane have a better antibiofilm effect. Curcumin, which acts by lysing the fungal cell, has a more powerful antibiofilm effect than piperine [17].

The biofilm formation enhances tolerance to antifungal drugs among *Candida* species and has necessitated the search for a new antifungal treatment strategy. Interference in pathogenic biofilm development by new antifungal compounds is considered an attractive antiinfective strategy [18]. This study evaluated the biofilm's abilities compared to the reference strain ATCC 9002 presented in Figure 1. The results showed that all our isolates formed biofilm at 48 hours with different percentages. The percentages of biofilm in the isolates were higher than for the reference strain ATCC 9002. The ability of *C. albicans* to switch morphology and form biofilms is the central property of their pathogenesis. Because biofilms formed by *C. albicans* are inherently tolerant of immune systems and conventional antifungals, and therefore, their susceptibility to current therapeutic agents remains low [19].

In the present study, a plant-derived alkaloid, piperine, polyphenol, and curcumin, were investigated for antibiofilm activity against *C. albicans* and presented in Table 2. Curcumin and piperine were effective against *C. albicans* biofilms. However, curcumin showed better activity against *C. albicans* biofilm compared to piperine. According to the R (MBEC/MBEC) ratio, the concentration of antifungal or natural substances for inhibition was lower than that of the eradicated biofilm of *Candida albicans*. In fact, by their mechanism of action, curcumin binds to ergosterol present in the membrane, which leads to fungal cell disruption and loss of intracellular content [20]. Piperine significantly downregulates the expression of several biofilm-related and hyphal-specific genes (ALS3, HWP1, EFG1, and CPH1) [21].

In addition to complete inhibition and eradication of biofilm, another strategy is to find combinations of compounds with anticandida activity [2]. The effect of the combination of antifungals and natural substances was evaluated, and the results are presented in Table 3. Our results showed that curcumin and piperine enhanced the activities of antifungals and presented a synergistic effect against *C. albicans*.

Curcumin combined with nystatin showed three synergistic effects against *C. albicans* strains, reducing 4-fold, 64-fold, and 4-fold, respectively, the MIC of nystatin. Moreover, combined with amphotericin B, it showed three synergistic effects, reducing 4-fold the MIC of amphotericin B. Two synergistic effects were also obtained with a combination of curcumin and fluconazole, reducing 8-fold and 32-fold the MIC of fluconazole. Then, combined with amphotericin B, it showed one synergistic effect and reduced 4-fold the MIC value of amphotericin B. No synergistic effects were obtained with a combination of curcumin and clotrimazole. Our results corroborated the previous studies, which reported the synergistic effect of all combinations of curcumin and amphotericin B, whereas both synergistic and additive effects were observed in the combination of curcumin and fluconazole, suggesting that these combinations should provide greater fungicidal effects for the treatment of systemic and superficial candidiasis [22].

As concerning piperine, in combination with fluconazole, it showed five synergistic effects against *C. albicans* isolates. Four synergistic effects were obtained with a combination of piperine and clotrimazole, with FICI values of 0.28, 0.37, 0.37, and 0.31, respectively, and reducing MIC values of fluconazole 32-fold, 4-fold, 8-fold, and 16-fold. Three synergistic effects were also reported with piperine and nystatin against isolates with FICI values ranging from 0.15 to 0.37 and reducing MIC values of nystatin from 8 to 64-fold. Two synergistic effects were shown with piperine and amphotericin B with FICI values of 0.25 and 0.5, respectively, and reducing the MIC value of amphotericin B. In the same idea, the synergistic effect of the combination of piperine with azoles (ketoconazole) was previously reported against *C. albicans* [23].

Among all our isolates and strains, piperine presented a double number (thirteen) of synergistic effects compared to curcumin (six) in combination with all antifungals. Curcumin presented more synergistic effects (four) combined with polyenes (amphotericin B and nystatin) compared to azoles (two) (clotrimazole and fluconazole). However, piperine presented more synergistic effects combined with azoles (nine) compared to polyenes (five).

The limitations of this study are threefold: firstly, we did not evaluate the mechanism of action at the molecular level of our synergistic combinations on the biofilm extracellular matrix. Furthermore, we did not evaluate the effect of the combinations on quorum sensing inhibition, a signaling mechanism that bacteria within the biofilm use to enhance their pathogenicity. Finally, in this study, synergistic combinations were obtained only in vitro and were not evaluated in vivo.

Overall, the difference between this study from similar ones lies in its comprehensive exploration of both curcumin and piperine, as well as their synergistic effects with antifungals. While previous studies have focused on other bacterial species, the inclusion of both curcumin and piperine in this research adds a layer of complexity that mirrors the potential multifaceted nature of combating *C. albicans* infections. This comprehensive approach enhances the translational potential of the study's findings, offering a more holistic strategy for clinicians and researchers to consider in the development of antifungal therapies. In summary, this study's strength lies in its unique focus on the synergistic potential of curcumin and piperine with antifungals against multiresistant *C. albicans* clinical isolates. The comprehensive exploration of these natural compounds and their combined effects sets this research apart from similar studies, providing a promising avenue for the development of innovative and effective antifungal strategies in clinical settings.

## 5. Conclusion

Candidiasis is a major life-threatening disease due to the increased incidence of drug resistance in *Candida* spp. and the limited antifungals available. *C. albicans* isolates were mostly resistant to azole antifungals compared to polyenes. Curcumin and piperine showed, respectively, significant and moderate activity against planktonic *C. albicans*. The resistance of *C. albicans* was mostly associated with biofilm formation. Antibiofilm and combination therapy may be a valid alternative. Natural substances curcumin and piperine showed antibiofilm activity, inhibition, and eradication of biofilm-multiresistant *C. albicans* isolates. The combination therapy showed a synergistic interaction between curcumin and piperine with antifungal polyenes and azoles against resistant *C. albicans*. There are many reports available on the combination of antifungal drugs with synthetic small molecules and with natural compounds in vitro. Some combinations were tested in vivo. There is a need to try these combinations in vivo.

## Figures and Tables

**Table 1:** Minimum inhibitory concentrations (MICs) of antifungals and natural substances against *C. albicans* strains.

Isolates	Minimum inhibitory concentrations (MIC, *μ*g/mL)					
	Antifungals				Natural substances	
	AmB	Nys	Clo	Flu	Curcumin	Piperine
ATCC 9002	32 (R)	64 (R)	128 (R)	128 (R)	128 (L)	512 (L)
*Ca01*	0.25 (S)	1 (S)	1 (I)	1 (S)	16 (M)	128 (L)
*Ca02*	0.25 (S)	32 (R)	16 (R)	128 (R)	32 (M)	512 (L)
*Ca03*	2 (I)	16 (R)	32 (R)	128 (R)	32 (M)	32 (M)
*Ca04*	0.5 (S)	0.5 (S)	16 (R)	0.5 (S)	256 (L)	512 (L)
*Ca05*	4 (I)	8 (R)	64 (R)	128 (R)	128 (L)	256 (L)
*Ca06*	64 (R)	64 (R)	16 (R)	—	256 (L)	1024 (N)
*Ca07*	64 (R)	128 (R)	0.125 (S)	128 (R)	0.125 (Si)	512 (L)
*Ca08*	1 (S)	4 (R)	8 (R)	—	64 (M)	128 (L)
*Ca09*	1 (S)	2 (I)	32 (R)	4 (S)	16 (M)	32 (M)
*Ca10*	64 (R)	64 (R)	4 (R)	64 (R)	128 (M)	128 (L)
*Ca11*	0.25 (S)	0.5 (S)	32 (R)	8 (S)	256 (L)	32 (M)
*Ca12*	1 (S)	0.5 (S)	2 (I)	16 (I)	256 (L)	128 (L)
*Ca13*	64 (R)	128 (R)	2 (I)	128 (R)	16 (M)	128 (L)
*Ca14*	1 (S)	32 (R)	32 (R)	32 (I)	256 (L)	512 (L)
*Ca15*	32 (R)	4 (I)	64 (R)	32 (I)	64 (M)	1024 (N)
*Ca16*	32 (R)	64 (R)	16 (R)	8 (S)	16 (M)	256 (L)
*Ca17*	—	0.25 (S)	1 (I)	1 (S)	256 (L)	128 (L)
*Ca18*	32 (R)	32 (R)	64 (R)	64 (R)	128 (L)	256 (L)
*Ca19*	32 (R)	4 (I)	8 (R)	16 (I)	256 (L)	512 (L)
*Ca20*	0.125 (S)	1 (S)	16 (R)	2 (S)	8 (Si)	64 (M)
MIC_50_	32	32	16	64	128	256
MIC_90_	64	64	32	128	256	512

MIC: minimum inhibitory concentration (*μ*g/mL); AmB: amphotericin B; Nys: nystatin; Clo: clotrimazole; Flu: fluconazole; Cur: curcumin; Pip: piperine; C.a: *Candida albicans*; ATCC 9002: American Type Culture Collection strain; (—): MIC of antifungals up to 128 *μ*g/mL; R: resistant; I: intermediate; S: susceptible; Si: significant activity; M: moderate activity; L: low activity; N: no activity; MIC50: concentration required to inhibit 50% of isolates; MIC90: concentrations required to inhibit 90% of isolates; the criteria (reference) used to define the activity of natural compounds as significant (Si), moderate (M), or low (L), or no activity (N) were defined as previously described [9].

**Table 2:** Antibiofilm activities of natural substances and antifungals against *Candida albicans*.

Isolates		Antifungals				Natural substances	
		AmB	Nys	Clo	Flu	Curcumin	Piperine
ATCC 9002	MBIC	1024	512	64	512	128	1024
	MBEC	—	—	—	1024	512	—
	R	nd	nd	nd	2	4	nd

*Ca02*	MBIC	128	128	128	256	256	1024
	MBEC	128	256	256	256	—	512
	R	1	2	2	1	nd	0.5

*Ca03*	MBIC	16	32	64	64	64	128
	MBEC	256	256	512	256	256	2048
	R	16	8	8	4	4	16

*Ca10*	MBIC	32	16	128	128	256	256
	MBEC	256	256	256	—	512	512
	R	8	16	2	nd	2	2

*Ca13*	MBIC	16	16	64	64	1024	128
	MBEC	256	256	256	—	—	512
	R	16	16	4	nd	nd	4

*Ca14*	MBIC	16	16	16	128	256	256
	MBEC	128	128	256	256	256	256
	R	8	8	16	2	1	1

*Ca16*	MBIC	16	64	8	16	128	128
	MBEC	128	64	256	256	256	256
	R	8	1	32	16	2	2

MBIC: minimum biofilm inhibitory concentration (*μ*g/mL); MBEC: minimum eradicated concentration (*μ*g/mL); AmB: amphotericin B; Nys: nystatin; Clo: clotrimazole; Flu: fluconazole; Cur: curcumin; Pip: piperine; C.a: *Candida albicans*; ATCC: American Type Culture Collection strain; (—): MBEC up to 128 *μ*g/mL for antifungals and up to 2048 *μ*g/mL for natural substances; R: MBEC/MBIC ratio; nd: not determined.

**Table 3:** Minimum inhibitory concentration (MIC) and effects of the combination of curcumin/piperine with amphotericin B, nystatin, clotrimazole, and fluconazole on *C. albicans* isolates.

Isolates	ATF	MIC (*μ*g/mL)							MIC reduction fold (antifungals)		FICI/INT	
		Alone			Combined							
		ATF	Cur	Pip	ATF/Cur	Cur	ATF/Pip	Pip	ATF/Cur	ATF/Pip	ATF/Cur	ATF/Pip
ATCC 9002	AmB	32	128	512	32	256	128	128	1	0.25	3/Ind	4/Ant
	Nys	64	128	512	16	32	2	64	4	32	0.5/Syn	0.15/Syn
	Clo	128	128	512	32	64	8	128	4	16	0.75/Add	0.31/Syn
	Flu	128	128	512	64	64	0.25	128	2	512	1/Add	0.5/Syn

*Ca02*	AmB	0.25	32	512	8	128	0.03	64	0.03	8	4.13/Ind	0.25/Syn
	Nys	32	32	512	4	16	16	128	8	2	0.62/Add	0.75/Add
	Clo	16	32	512	8	16	4	64	2	4	1/Add	0.37/Syn
	Flu	128	32	512	16	8	0.06	128	8	256	0.31Syn	0.31/Syn

*Ca03*	AmB	2	32	32	1	128	0.5	16	2	4	4.5/Ant	0.75/Syn
	Nys	16	32	32	8	32	128	64	2	0.12	1.5/Add	10/Ant
	Clo	32	32	32	8	64	1	8	4	32	2.25/Ind	0.28/Syn
	Flu	128	32	32	4	8	1	128	32	128	0.28/Syn	5/Ant

*Ca10*	AmB	64	128	128	16	64	128	64	4	0.5	0.75/Add	2.5/Ind
	Nys	64	128	128	16	64	128	64	4	0.5	0.75/Add	2.5/Ind
	Clo	4	128	128	1	64	2	64	4	2	0.75/Add	1/Add
	Flu	64	128	128	256	128	0.03	16	0.25	2048	5/Ant	0.15/Syn

*C 13*	AmB	64	16	128	64	32	32	512	1	2	3/Ind	4.5/Ant
	Nys	128	16	128	2	4	128	64	64	1	0.26/Syn	1.5/Ind
	Clo	2	16	128	8	64	0.5	128	0.25	4	8/Ant	1.25/Ind
	Flu	128	16	128	32	32	0.25	256	4	512	2.25/Int	2.25/Ind

*Ca14*	AmB	1	256	512	0.25	64	0.25	64	4	4	0.5/Syn	0.38/Syn
	Nys	32	256	512	8	64	4	128	4	8	0.5/Syn	0.37/Syn
	Clo	32	256	512	4	128	4	128	8	8	0.62/Add	0.37/Syn
	Flu	32	256	512	64	128	0.06	128	0.5	2048	2.5/Ind	0.31/Syn

*Ca16*	AmB	32	16	256	64	64	1	128	0.5	32	6/Ant	0.58/Add
	Nys	64	16	256	32	32	1	64	2	64	2.5/Ind	0.26/Syn
	Clo	16	16	256	8	32	8	64	2	2	2.5/Ind	0.75/Ind
	Flu	8	16	256	4	8	0.125	64	2	64	1/Add	0.37/Syn

MIC: minimum inhibitory concentration; Cur: curcumin; Pip: piperine; ATF: antifungals; ATF/Cur: a combination of antifungal with curcumin; ATF/Pip: a combination of antifungals with piperine; FICI: fractional inhibitory concentration index; INT: interpretation; Syn: synergy, Add: additivity; Ind: indifference; Ant: antagonism; C.a: *C. albicans* isolate; ATCC 9002: American Type Culture Collection strain. AMB: amphotericin B; NYS: nystatin, CLO: clotrimazole; FLU: fluconazole.

## Data Availability

The data used to support the findings of this study are available upon reasonable request from the corresponding author.
